# Association between Low-carbohydrate Diets and 10-year Atherosclerosis Cardiovascular Disease Risk: Data from the National Health and Nutrition Examination Survey

**DOI:** 10.2174/0115733998411207251130184836

**Published:** 2026-01-20

**Authors:** Yipeng Du, Hong Ouyang, Huiyong Wang, Jian Shi, Yuanqing Li, Huidong Long, Xiudi Pan

**Affiliations:** 1 Department of Cardiology, The First Affiliated Hospital of Guangzhou Medical University, Guangzhou, China;; 2 Pharmacy IntraVenous Admixture Service, The First Affiliated Hospital of Guangzhou Medical University, Guangzhou, China;; 3 The First Affiliated Hospital of Guangzhou University of Chinese Medicine, Guangzhou, China;; 4 Department of Medical Oncology, Affiliated Cancer Hospital and Institute of Guangzhou Medical University, Guangzhou, China

**Keywords:** Low-carbohydrate diets, atherosclerotic cardiovascular disease, type 2 diabetes mellitus, chronic kidney disease, high-density lipoprotein cholesterol, high fat may

## Abstract

**Introduction:**

To evaluate the association between low-carbohydrate diet (LCD) score and atherosclerotic cardiovascular disease (ASCVD) among individuals with type 2 diabetes mellitus (T2DM).

**Methods:**

A total of 3685 participants from the National Health and Nutrition Examination Survey database 2007-2020 were included in this cross-sectional study. The primary outcome of this study was the 10-year ASCVD risk. We used the weighted logistic regression models to assess the association between LCD score and 10-year ASCVD risk. Stratified analysis based on age, body mass index (BMI), hypertension, dyslipidemia, and chronic kidney disease (CKD) was conducted. Odds ratio (OR) and 95% confidence interval (CI) were calculated.

**Results:**

After adjusting for all confounding factors, when LCD was treated as a continuous variable, the findings revealed an inverse association between LCD and 10-year ASCVD risk among individuals with T2DM (OR=0.97, 95% CI: 0.94-0.99, *p*=0.045). When LCD was stratified into tertiles, individuals in the highest tertiles of LCD exhibited a negative association with 10-year ASCVD risk compared to those in the first tertile (OR=0.66, 95% CI: 0.46-0.95, *p*=0.025). Subgroup analysis indicated an negative association between high tertiles of LCD and 10-years ASCVD risk in individuals with T2DM who had a BMI≥25kg/m^2^ (OR=0.66, 95% CI: 0.45-0.97, *p*<0.05), hypertension (OR=0.61, 95% CI: 0.40-0.91, *p*<0.05) and dyslipidemia (OR=0.64, 95% CI: 0.43-0.95, *p*<0.05), and BMI<25kg/m^2^ with history of CKD (OR=0.64, 95% CI: 0.42-0.96, *p*<0.05).

**Discussion:**

The inverse association observed between LCD score and ASCVD risk in individuals with T2DM may be partly explained by improved glycemic control, reduced insulin resistance, and favorable lipid profile changes often associated with lower carbohydrate intake. Notably, the protective effect was more pronounced in individuals with additional metabolic comorbidities, such as obesity, hypertension, and dyslipidemia, suggesting that these subgroups may derive greater cardiovascular benefit from LCD-style eating patterns. However, the cross-sectional nature of our study precludes causal inference. Additionally, dietary intake was self-reported and subject to recall bias, and residual confounding cannot be fully ruled out. Despite these limitations, our findings contribute to the growing body of evidence supporting dietary carbohydrate modification as a potential strategy in cardiovascular risk management among patients with T2DM.

**Conclusion:**

Our study found an inverse association between LCD score and 10-year ASCVD risk in individuals with T2DM. These findings may provide a reference for future research and inform dietary recommendations, though causal relationships cannot be established due to the cross-sectional design.

## 1 INTRODUCTION

Atherosclerotic cardiovascular disease (ASCVD) remains a leading cause of global morbidity and mortality despite significant progress in the management of conventional risk factors [1]. Type 2 diabetes mellitus (T2DM) is widely acknowledged as a risk factor for ASCVD, with ASCVD being the primary cause of all-cause mortality and cardiovascular-related deaths in patients with T2DM [2, 3]. The increasing global aging population has led to a rise in the prevalence of T2DM and its associated ASCVD, resulting in a significant disease burden [4]. Therefore, exploring the impact of relevant modifiable factors for ASCVD among T2DM patients is important in reducing ASCVD mortality and alleviating the healthcare burden.

Dietary intervention has consistently served as an important means in the management of chronic diseases [5]. A heart-healthy dietary pattern was found to be an intervening measure for treating patients with T2DM, mitigating ASCVD risk through enhanced glycemic control and weight reduction [6, 7]. Increasing research suggested that low-carbohydrate diets (LCD) play a crucial role in the management of T2DM, primarily due to their potential impact on glycemic control through carbohydrate restriction [8-12]. However, some studies have reported conflicting findings regarding the impact of LCD on ASCVD risk, such as an observational case-control study that has indicated that dietary patterns characterized by high-fat and low-carbohydrate intake may be associated with an increased risk of ASCVD [13-15]. LCD may potentially contribute to the unrestrained consumption of saturated fat, resulting in elevated levels of low-density lipoprotein cholesterol (LDL-C), thereby increasing the risk of cardiovascular disease (CVD) [16, 17]. There is also evidence suggesting that LCD has no adverse effects on ASCVD and its associated risk factors [18]. A meta-analysis based on randomized controlled trials demonstrated that LCD intervention yielded favorable outcomes in terms of glycemic control, triglyceride (TG), and high-density lipoprotein cholesterol (HDL-C) levels among patients with T2DM. However, no significant effects on LDL-C were observed [19]. Based on this, we speculated that LCD might exert an impact on the susceptibility to ASCVD in individuals with T2DM.

However, as far as we know, the relationship between LCD and a 10-year ASCVD risk remains unclear. Therefore, this study was designed using the National Health and Nutrition Examination Survey (NHANES) dataset to investigate the association between LCD and a 10-year ASCVD risk among individuals with T2DM.

## 2 METHODS

### 2.1 Data Sources and Study Population

All data for this cross-sectional study were obtained from the NHANES database. NHANES, a complex, multistage, and probabilistic sampling design survey, combines interviews (demographic, socioeconomic, dietary, and health-related questions) with physical examinations (medical, dental, physiological measurements, and laboratory tests).

We initially identified 6007 individuals aged 40-79 years with a diagnosis of type 2 diabetes mellitus (T2DM) from the NHANES 2007-2020. The inclusion criteria were: (1) age between 40 and 79 years; (2) confirmed diagnosis of T2DM; (3) availability of dietary intake data from the 24-hour dietary recall interview; and (4) availability of all necessary variables for calculating the 10-year ASCVD risk score. Participants were excluded if they had: (1) a prior history of cardiovascular disease (self-reported myocardial infarction, stroke, or congestive heart failure; n = 1159); (2) missing data on dietary intake (n = 644); (3) missing data required for calculating 10-year ASCVD risk (n = 445); or (4) missing information on body mass index (BMI, kg/m^2^) (n=74). After applying these criteria, a total of 3685 eligible participants were included in the final analysis. The sample selection process is illustrated in Fig. (**1**). These criteria ensured the selection of a homogenous study population free of established cardiovascular disease and with complete baseline, dietary, and risk factor data.

### 2.2 Data Collection

#### 2.2.1 Assessment of the Predicted 10-year ASCVD Risk

The primary outcome of this study was the 10-year ASCVD risk, which was defined as the occurrence of a first non-fatal myocardial infarction (MI), death from coronary heart disease (CHD), or fatal or non-fatal stroke within 10 years. According to the American College of Cardiology and the American Heart Association in 2013 [20], this algorithm estimates the 10-year probability of a first hard ASCVD event, defined as nonfatal myocardial infarction, coronary heart disease death, or stroke. The calculation incorporates the following variables: age, sex, race (White, African American, or other), total cholesterol (mg/dL), HDL-C (mg/dL), systolic blood pressure (mmHg), current treatment for hypertension (yes/no), current smoking status (yes/no), and presence of diabetes (yes/no). We used these parameters extracted from NHANES to compute each participant’s risk using established formulae. Participants with a 10-year ASCVD risk score of ≥7.5% were classified as having a high-risk, while those with a score <7.5% were identified as low-risk individuals.

#### 2.2.2 Assessment of Low-carbohydrate Diets

The LCD score was derived from a comprehensive evaluation of fat, protein, and carbohydrate [21]. Fat, protein, and carbohydrate from the diet and supplements were expressed as a percentage of energy: Fat (%) = fat (g) *9/ total energy (kcal) *100; protein (%) = protein (g)*4/ total energy (kcal) *100; carbohydrate (%) = carbohydrate (g) *4/ total energy (kcal) *100. Fat intake score, protein intake score, and carbohydrate intake score were calculated based on “Criteria for Determining the Low-Carbohydrate Diet Score”. The LCD score ranged from 0 to 30, with higher scores indicating a higher fat and protein intake and lower carbohydrate intake. In this study, the LCD score was classified into three categories based on tertiles: ≤12 points, 12~17 points, and >17 points. The LCD score was calculated based on dietary intake data obtained from a single 24-hour dietary recall interview in NHANES.

#### 2.2.3 Assessment of Possible Covariates

We included a range of covariates to control for potential confounding factors. Continuous variables included age, body mass index (BMI), fiber intake, and LCD score. Categorical variables were classified as follows: gender (male/female), race (non-Hispanic White, non-Hispanic Black, others), and marital status (married or living with a partner, widowed/divorced/separated, never married). Hypertension was defined according to the 2024 ESC Hypertension Guidelines as a self-reported physician diagnosis, systolic blood pressure ≥130 mmHg, and/or diastolic blood pressure ≥80 mmHg, or the use of antihypertensive medication [22]. Dyslipidemia was classified based on the 2019 ESC Dyslipidaemias Guidelines, with criteria including total cholesterol ≥200 mg/dL, triglycerides ≥150 mg/dL, LDL-C ≥130 mg/dL, HDL-C ≤40 mg/dL, or the use of lipid-lowering medication [23]. Additional covariates included chronic kidney disease (CKD) (yes, defined as a self-reported diagnosis or estimated glomerular filtration rate <60 mL/min/1.73m^2^; no), smoking status (never smoker, former smoker, current smoker), drinking status (never, less than once per week, ≥ once per week), diabetic retinopathy (yes, no, unknown), family history of cardiovascular disease (CVD) (yes, no), and duration of diabetes (≤5 years, >5 years, unknown).

### 2.3 Statistical Analysis

Given the complex sampling design of NHANES, all analyses utilized the appropriate data weights for NHANES samples. SDMVPSU (Primary Sampling Unit), SDMVSTRA (Stratum), WTMEC2YR (2-Year Examination Cycle Weight), and WTMECPRP (Mobile Examination Center Participation Weight), which account for the complex sampling design of NHANES and ensure that the data are representative of the general U.S. population, were used as weighted variables in this study. Continuous variables were represented by the weighted mean ± standard error (SE), and a t-test was used to compare the groups. The number of cases and the composition ratio [n (%)] were used to describe the categorical data, and a comparison between two groups adopted the Chi-square test.

Weighted univariate logistic regression analysis was used to screen the confounding variables related to 10-year ASCVD risk in this analysis (Table **S1**, *p*<0.05). Subsequently, we developed the weighted logistic regression models to assess the association between LCD score and 10-year ASCVD risk among individuals with T2DM. Odds ratio (OR) and 95% confidence interval (CI) were calculated. Additional subgroup analyses were also performed to explore whether the impact of LCD score on 10-year ASCVD risk differed across these subsets, stratifying by BMI, hypertension, dyslipidemia, and CKD. For the variables with missing data, we employed multiple interpolation methods based on a chained equation approach and conducted sensitivity analysis on the data before and after interpolation (Table **S2**). *p*<0.05 was considered statistically significant. All analyses were performed using SAS 9.4 software (SAS Institute Inc., Cary, NC, USA).

## 3 RESULTS

### 3.1 Characteristics of Participants

A total of 3685 individuals with T2DM were included in this study, with a mean age of 58.79 (0.26) years. The study comprised 1852 male and 1833 female participants, and the average LCD score was 15.00 (0.11) points. The characteristics of the participants with T2DM, stratified by 10-year ASCVD risk, are shown in Table **1**, from which we found statistically significant differences in age, sex, race, marital status, hypertension, dyslipidemia, CKD, duration of diabetes, and BMI (all *p*<0.05). Compared to participants with 10-year ASCVD risk <7.5%, those with 10-year ASCVD risk ≥7.5% were more likely to be older, male, widowed/divorced/separated, and had a higher prevalence of hypertension, dyslipidemia, and CKD, as well as demonstrated lower levels of BMI.

### 3.2 Association between LCD and 10-year ASCVD Risk

We employed weighted logistic regression models to investigate the relationship between LCD and 10-year ASCVD risk among individuals with T2DM. As shown in Table **2**, when LCD was treated as a continuous variable, and after adjusting for age, sex, race, marital status, drinking status, hypertension, dyslipidemia, CKD, and duration of diabetes, the findings revealed an negative association between LCD and 10-years ASCVD risk among individuals with T2DM (OR=0.97, 95% CI: 0.94-0.99, *p*=0.045). Subsequently, when LCD was stratified into tertiles, the highest tertiles of LCD demonstrated a reduced risk of 10-year ASCVD (Model 1: OR=0.67, 95% CI: 0.47-0.98, *p*=0.038). In the fully adjusted model (Model 2), individuals in the highest tertiles of LCD exhibited a negative association with 10-year ASCVD risk compared to those in the first tertile (OR=0.66, 95% CI: 0.46-0.95, *p*=0.025).

### 3.3 Subgroup Analysis

Subgroup analysis stratified by BMI, hypertension, dyslipidemia, and CKD was performed. As shown in Table **3** and Fig. (**2**), after adjusting for all confounding variables, the results indicated an negative association between high tertiles of LCD and 10-years ASCVD risk in individuals with T2DM who had a BMI≥25kg/m^2^ (OR=0.66, 95% CI: 0.45-0.97, *p*=0.033), hypertension (OR=0.61, 95% CI: 0.40-0.91, *p*=0.017) and dyslipidemia (OR=0.64, 95% CI: 0.43-0.95, *p*=0.026), and BMI<25kg/m^2^ with history of CKD (OR=0.64, 95% CI: 0.42-0.96, *p*=0.033).

## 4 DISCUSSION

This study assessed the association of LCD score and 10-year ASCVD risk among individuals with T2DM based on a large sample from the NHANES database. After adjusting for relevant confounding variables, regardless of whether it is considered as a continuous or categorical variable, the LCD score was found to be associated with decreased odds of 10-year ASCVD risk. The findings suggested the importance of low-carbohydrate diet patterns in the context of ASCVD for populations with T2DM.

Recently, the LCD pattern has been proposed and considered beneficial to health [24, 25]. For patients with T2DM, reducing dietary carbohydrate intake has been suggested as a recommended approach to enhance glycemic control and optimize outcomes [26]. A systematic review and meta-analysis revealed that adhering to LCD for six months might attain greater rates of diabetes remission without adverse consequences [27]. Systematic review also demonstrated that a low-carbohydrate diet pattern was a potentially effective treatment option for individuals with diabetes or obesity [28]. Additionally, numerous studies are currently examining the impact of a low-carbohydrate diet on ASCVD. Meta-analysis indicated that low-carbohydrate diets significantly lower predicted risk of ASCVD events [29]. The diet pattern of a high-fat, low-carbohydrate consumption was found to be associated with an elevated risk of ASCVD [13]. However, as far as we know, the potential impact of adhering to an LCD pattern on the reduction of ASCVD risk among individuals with diabetes remains unclear. In this cross-sectional study, we utilized the assessment equation based on the American College of Cardiology and the American Heart Association in 2013 to estimate the 10-year risk of a first ASCVD event and found that the LCD score was significantly associated with a lower odds of 10-year ASCVD risk for individuals with T2DM. In other words, adhering to a dietary pattern characterized by higher fat and protein intake along with lower carbohydrate intake may confer potential benefits in reducing the incidence of ASCVD in diabetic patients. The possible explanations were: ASCVD is a chronic inflammatory disease. Studies have shown that LCD may be associated with greater reductions in inflammatory markers, body fat mass, fasting glucose levels, triglycerides, and blood pressure compared to low-fat meals [30, 31]. A controlled trial also demonstrated that a reduction in dietary carbohydrate intake significantly improved some pro-inflammatory markers, such as interleukin-6, interleukin-8, and Tumour Necrosis Factor alpha [32]. The maintenance of healthy blood vessels depends on the presence of healthy endothelial cells [33]. However, in the context of anterior arteriosclerosis, these endothelial cells change to a pathogenic phenotype, ultimately damaging the vessel wall. As a result, endothelial dysfunction has been widely recognized as the main cause of atherosclerosis and CVD [34, 35]. Acute impairment of the endothelial vasodilatory system is exacerbated by hyperglycemia caused by high carbohydrate intake and increased oxidative stress [36]. Additional evidence in the future is imperative for a more comprehensive comprehension of intricate biological mechanisms.

Through further subgroup analysis, we found that the negative association between LCD score and 10-year ASCVD risk was significant in the individuals with T2DM who had a BMI≥25kg/m^2^, hypertension and dyslipidemia, and no history of CKD. In individuals without CKD, the inverse association between LCD score and 10-year ASCVD risk is more pronounced, suggesting that a dietary pattern characterized by higher fat and protein intake, along with lower carbohydrate intake, confers significant benefits in T2DM participants without CKD. Studies have shown that high protein and high fat may accelerate CKD progression [37-39].

From our perspective, these findings underscore the complexity of dietary interventions in cardiometabolic disease prevention. While a low-carbohydrate pattern may improve glycemic control and reduce inflammation, the overall impact likely depends on the quality and source of macronutrients. Our study contributes to the ongoing discussion by providing nationally representative data supporting a potential benefit of LCDs in T2DM populations. However, individual dietary recommendations should remain personalized, balancing benefits and risks.

To the best of our knowledge, this study is the first to investigate the relationship between LCD score and 10-year ASCVD risk in individuals with T2DM in the United States. The large sample size and complex sampling methodology employed from the general population enable extrapolation to the U.S. population. However, some limitations should be taken into account. First, due to the cross-sectional nature of this study, we are unable to infer causality between LCD adherence and ASCVD risk reduction. Second, despite adjustment for multiple covariates, residual confounding cannot be ruled out, as factors such as detailed socioeconomic status, overall dietary quality, and lifestyle behaviors may not have been fully captured. Third, some of the observed associations, although statistically significant, had odds ratios close to 1.0, indicating that the clinical magnitude of the effect may be modest. Fourth, our subgroup analyses were exploratory in nature and involved multiple hypothesis tests. Because we did not apply formal multiple testing corrections, these findings should be interpreted cautiously, and considered hypothesis-generating rather than confirmatory. Fifth, while subgroup stratification was performed, we were unable to conduct formal interaction testing due to dataset and analytic limitations; therefore, the observed subgroup differences may be due to chance. Sixth, our analysis assumed a linear relationship between LCD score and ASCVD risk. Although this assumption facilitated interpretation, we were unable to model potential nonlinear associations, which should be examined in future studies with richer dietary data. Finally, as this was a cross-sectional study, causal inference cannot be established, and prospective studies or randomized controlled trials will be required to validate our findings.

## CONCLUSION

In this cross-sectional analysis of nationally representative NHANES data, a higher low-carbohydrate diet (LCD) score was inversely associated with estimated 10-year ASCVD risk among individuals with type 2 diabetes mellitus. This association remained consistent across multiple adjusted models and appeared more pronounced in participants with coexisting metabolic conditions, including overweight or obesity, hypertension, and dyslipidemia. These findings suggest that dietary patterns characterized by lower carbohydrate intake and relatively higher fat and protein intake may be linked to a more favorable cardiovascular risk profile in this population. Nevertheless, due to the observational and cross-sectional nature of the study, causal inferences cannot be established. Future prospective studies and randomized controlled trials are warranted to confirm these associations and to clarify the long-term cardiovascular effects and optimal composition of low-carbohydrate dietary patterns in patients with type 2 diabetes.

## Figures and Tables

**Fig. (1):**
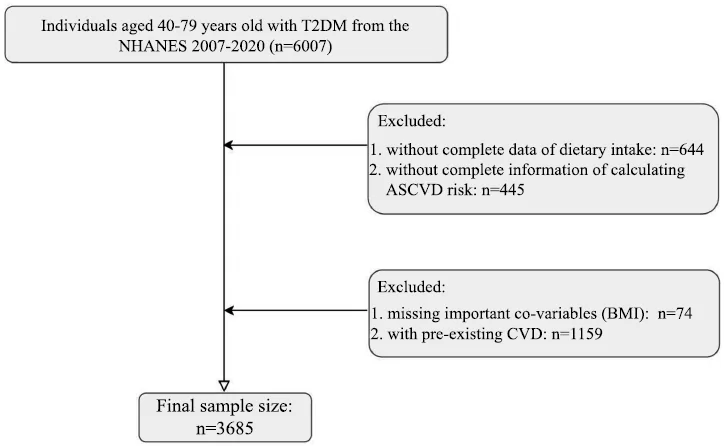
Flow chart of study participants.

**Fig. (2):**
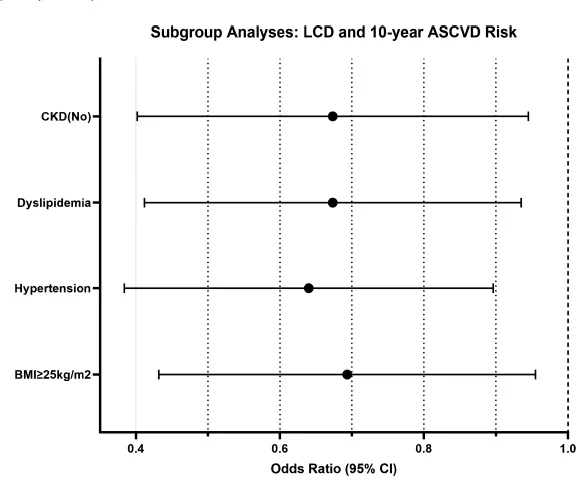
Subgroup Analyses:LCD and 10-year ASCVD Risk.

**Table 1:** Characteristics of participants.

**Variables**	**Total (n=3685)**	**Low 10-year ASCVD risk** **(n=756)**	**High 10-year ASCVD risk (n=2929)**	***p***
**LCD score, Mean (S.E)**	15.00 (0.11)	15.25 (0.27)	14.93 (0.12)	0.293
**LCD score, n (%)**				0.304
≤12	1413 (32.80)	271 (30.57)	1142 (33.48)	-
12~17	1236 (33.84)	268 (32.86)	968 (34.14)	-
>17	1036 (33.36)	217 (36.56)	819 (32.38)	-
Age, years, Mean (S.E)	58.79 (0.26)	48.86 (0.33)	61.81 (0.25)	<0.001
**Age, n (%)**				<0.001
<60 years	1638 (52.76)	712 (95.37)	926 (39.78)	-
≥60 years	2047 (47.24)	44 (4.63)	2003 (60.22)	-
**Sex, n (%)**				<0.001
Male	1852 (51.43)	204 (33.29)	1648 (56.96)	-
Female	1833 (48.57)	552 (66.71)	1281 (43.04)	-
**Race, n (%)**				<0.001
Non-Hispanic White	1087 (60.08)	190 (52.85)	897 (62.28)	-
Non-Hispanic Black	993 (14.20)	235 (18.61)	758 (12.85)	-
Others	1605 (25.72)	331 (28.54)	1274 (24.87)	-
**Education, n (%)**				0.226
Below senior high school	1183 (20.60)	205 (18.45)	978 (21.25)	-
Senior high school	825 (24.82)	159 (23.06)	666 (25.36)	-
University and above	1677 (54.58)	392 (58.49)	1285 (53.39)	-
**Marital status, n (%)**				0.025
Married/Living with Partner	2310 (67.52)	481 (70.32)	1829 (66.67)	-
Widowed/Divorced/Separated	1047 (24.54)	172 (20.21)	875 (25.86)	-
Never married	328 (7.94)	103 (9.47)	225 (7.47)	-
**PIR, n (%)**				0.586
≤1.3	1221 (21.99)	253 (22.03)	968 (21.98)	-
1.3~3.5	1467 (37.44)	289 (35.37)	1178 (38.07)	-
>3.5	997 (40.57)	214 (42.60)	783 (39.95)	-
**Drinking, n (%)**				0.112
No	1268 (29.97)	303 (33.41)	965 (28.92)	-
Less than once a week	1700 (48.74)	329 (47.86)	1371 (49.01)	-
Greater than or equal to once a week	717 (21.29)	124 (18.73)	593 (22.07)	-
**Physical activity, n (%)**				0.128
Insufficient physical activity	582 (15.72)	124 (17.87)	458 (15.07)	-
Sufficient physical activity	1808 (52.33)	397 (54.47)	1411 (51.67)	-
Unknown	1295 (31.95)	235 (27.66)	1060 (33.26)	-
**Hypertension, n (%)**				<0.001
No	854 (24.16)	332 (43.50)	522 (18.27)	-
Yes	2831 (75.84)	424 (56.50)	2407 (81.73)	-
**Dyslipidemia, n (%)**				<0.001
No	413 (10.72)	151 (19.19)	262 (8.14)	-
Yes	3272 (89.28)	605 (80.81)	2667 (91.86)	-
**Diabetic retinopathy, n (%)**				0.565
No	2026 (55.88)	415 (53.98)	1611 (56.45)	-
Yes	457 (10.94)	85 (12.30)	372 (10.52)	-
Unknown	1202 (33.19)	256 (33.72)	946 (33.03)	-
**CKD, n (%)**				<0.001
No	2654 (75.15)	620 (85.42)	2034 (72.02)	-
Yes	1031 (24.85)	136 (14.58)	895 (27.98)	-
**Family history of CVD, n (%)**				0.515
No	3168 (83.61)	633 (82.52)	2535 (83.94)	-
Yes	517 (16.39)	123 (17.48)	394 (16.06)	-
**Duration of diabetes, n (%)**				0.006
≤5	888 (25.43)	229 (30.66)	659 3.84)	-
>5	1599 (41.49)	272 (35.79)	1327 (43.22)	-
Unknown	1198 (33.08)	255 (33.55)	943 (32.94)	-
BMI, kg/m^2^, Mean (S.E)	33.02 (0.18)	34.36 (0.35)	32.62 (0.19)	<0.001
**BMI, n (%)**				0.682
<25kg/m^2^	440 (9.64)	85 (10.16)	355 (9.48)	-
≥25kg/m^2^	3245 (90.36)	671 (89.84)	2574 (90.52)	-
Fiber, g, Mean (S.E)	17.14 (0.28)	16.61 (0.66)	17.30 (0.32)	0.351
**Drug for diabetes, n (%)**				0.268
No	1293 (35.16)	290 (37.46)	1003 (34.46)	-
Yes	2392 (64.84)	466 (62.54)	1926 (65.54)	-

**Abbreviations:** ASCVD=atherosclerotic cardiovascular disease; LCD=low-carbohydrate diets; PIR=poverty to income ratio; CKD=chronic kidney disease; CVD=cardiovascular disease; BMI=body mass index.

**Table 2:** Association between LCD score and 10-year ASCVD risk.

**Variables**	**Model 1**		**Model 2**	
	**OR (95% CI)**	***p***	**OR (95% CI)**	***p***
LCD score	0.97 (0.94-1.00)	0.066	0.97 (0.94-0.99)	0.045
LCD score	-	-	-	-
≤12	Ref	-	Ref	-
12~17	1.01 (0.72-1.43)	0.951	1.04 (0.71-1.50)	0.850
>17	0.67 (0.47-0.98)	0.038	0.66 (0.46-0.95)	0.025

**Abbreviations:** ASCVD=atherosclerotic cardiovascular disease; LCD=low-carbohydrate diets; OR=odds ratio; CI=confidence interval; Model 1: adjusted age, sex, race, and marital status; Model 2: adjusted age, sex, race, marital status, drinking status, hypertension, *dyslipidemia*, chronic kidney disease, and duration of diabetes.

**Table 3:** Subgroup analyses.

**Variables**	**OR (95%CI)**	***p***	**OR (95%CI)**	***p***
**Subgroup I: BMI**	**BMI<25kg/m^2^ (n= 440)**		**BMI≥25kg/m^2^ (n= 3245)**	
LCD score	0.95 (0.86-1.04)	0.241	0.97 (0.94-1.00)	0.060
LCD score				-
≤12	Ref		Ref	-
12~17	2.09 (0.77-5.71)	0.147	1.01 (0.69-1.48)	0.949
>17	0.53 (0.20-1.42)	0.201	0.66 (0.45-0.97)	0.033
**Subgroup II: Hypertension**	No (n= 854)		Yes (n= 2831)	
LCD score	0.97 (0.92-1.02)	0.234	0.97 (0.94-1.01)	0.098
LCD score				-
≤12	Ref		Ref	-
12~17	1.37 (0.69-2.72)	0.367	0.94 (0.60-1.48)	0.785
>17	0.79 (0.42-1.49)	0.469	0.61 (0.40-0.91)	0.017
**Subgroup III: Dyslipidemia**	No (n= 413)		Yes (n= 3272)	
LCD score	0.98 (0.91-1.05)	0.557	0.97 (0.94-1.00)	0.058
LCD score				-
≤12	Ref		Ref	-
12~17	2.23 (0.81-6.12)	0.119	0.99 (0.67-1.47)	0.973
>17	0.94 (0.43-2.09)	0.885	0.64 (0.43-0.95)	0.026
**Subgroup IV: CKD**	No (n=2654)		Yes (n=1031)	
LCD score	0.97 (0.93-0.99)	0.039	1.01 (0.94-1.07)	0.833
LCD score				-
≤12	Ref		Ref	-
12~17	1.10 (0.73-1.65)	0.654	0.80 (0.37-1.71)	0.553
>17	0.64 (0.42-0.96)	0.033	0.86 (0.36-2.04)	0.722

**Abbreviations:** ASCVD=atherosclerotic cardiovascular disease; LCD=low-carbohydrate diets; OR=odds ratio; CI=confidence interval; CKD=chronic kidney disease; Adjusted age, sex, race, marital status, drinking status, hypertension (not adjusted in the Subgroup II), dyslipidemia (not adjusted in the Subgroup III), CKD (not adjusted in the Subgroup IV), and duration of diabetes.

## Data Availability

The data that support the findings of this study are publicly available from the National Health and Nutrition Examination Survey (NHANES) at https://www.cdc.gov/nchs/nhanes/.

## LIST OF ABBREVIATIONS

LCD: Low-carbohydrate Diets
ASCVD: Atherosclerotic Cardiovascular Disease
T2DM: Type 2 Diabetes Mellitus
TG: Triglyceride
HDL-C: High-density Lipoprotein Cholesterol

## STANDARDS OF REPORTING

STROBE guidelines were followed.
