# Supplementary material for: Association between Low-carbohydrate Diets and 10-year Atherosclerosis Cardiovascular Disease Risk: Data from the National Health and Nutrition Examination Survey
Source: Curr Diabetes Rev. 2026 Jan 20;22:E15733998411207. doi: 10.2174/0115733998411207251130184836 (PMC13714736; doi:10.2174/0115733998411207251130184836)
Supplement: Supplementary file 1 [file CDR-22-6-E15733998411207_SD1.pdf]

Supplementary Material

Association between Low-carbohydrate Diets and 10-year Atherosclerosis Cardiovascular Disease Risk: Data from the National Health and Nutrition Examination Survey

Yipeng Du[1,#], Hong Ouyang[2,#], Huiyong Wang[1], Jian Shi[1], Yuanqing Li[1], Huidong Long[3,4,*] and Xiudi Pan[1,*]

[1]*Department of Cardiology, The First Affiliated Hospital of Guangzhou Medical University, Guangzhou, China;* [2]*Pharmacy IntraVenous Admixture Service, The First Affiliated Hospital of Guangzhou Medical University, Guangzhou, China;* [3]*The First Affiliated Hospital of Guangzhou University of Chinese Medicine, Guangzhou, China;* [4]*Department of Medical Oncology, Affiliated Cancer Hospital and Institute of Guangzhou Medical University, Guangzhou, China*

Table S1. Confounding variables related to 10-year ASCVD risk.

**Variables**	**OR (95%CI)**	***P***
Age	1.22 (1.20-1.24)	<0.001
Gender		
Male	Ref	
Female	0.38 (0.29-0.49)	<0.001
Race		
Non-Hispanic White	Ref	
Non-Hispanic Black	0.59 (0.46-0.75)	<0.001
Others	0.74 (0.58-0.94)	0.013
Education, n (%)		
Below senior high school	Ref	
Senior high school	0.95 (0.67-1.36)	0.797
University and above	0.79 (0.62-1.01)	0.062
Marital status		
Married/Living with Partner	Ref	
Widowed/Divorced/Separated	1.35 (1.04-1.76)	0.027
Never married	0.83 (0.58-1.20)	0.319
PIR		
≤1.3	Ref	

1.3~3.5	1.08 (0.84-1.39)	0.549
>3.5	0.94 (0.71-1.25)	0.664
Drinking		
No	Ref	
Less than once a week	1.18 (0.93-1.50)	0.167
Greater than or equal to once a week	1.36 (1.00-1.86)	0.051
Physical activity		
Insufficient physical activity	Ref	
Sufficient physical activity	1.12 (0.78-1.62)	0.524
Unknown	1.43 (0.95-2.14)	0.086
Hypertension		
No	Ref	
Yes	3.44 (2.69-4.41)	<0.001
Dyslipidemia		
No	Ref	
Yes	2.68 (1.90-3.78)	<0.001
Diabetic retinopathy		
No	Ref	
Yes	0.82 (0.53-1.26)	0.354
Unknown	0.94 (0.74-1.18)	0.579
CKD		
No	Ref	
Yes	2.27 (1.69-3.06)	<0.001
Family history of CVD		
No	Ref	
Yes	0.90 (0.66-1.23)	0.517
Duration of diabetes		
≤5	Ref	

>5	1.55 (1.16-2.08)	0.003
Unknown	1.26 (0.98-1.62)	0.067
BMI		
<25kg/m^2	Ref	
≥25kg/m^2	1.08 (0.74-1.57)	0.683
Fiber	1.01 (0.99-1.02)	0.386
Drug for diabetes		
No	Ref	
Yes	1.14 (0.90-1.44)	0.269

ASCVD=atherosclerotic cardiovascular disease; LCD=low-carbohydrate diets; PIR=poverty to income ratio; CKD=chronic kidney disease; CVD=cardiovascular disease; BMI=body mass index; OR=odds ratio; CI=confidence interval.

Table S2. Sensitivity analysis on the data before and after interpolation.

**Variables**	**Before interpolation**	**After interpolation**	**Statistics**	***P***
Education, n (%)			χ^2=1.12	0.572
Below senior high school	1182 (20.60)	1183 (20.60)		
Senior high school	825 (24.83)	825 (24.82)		
University and above	1676 (54.57)	1677 (54.58)		
Marital status, n (%)			χ^2=1.98	0.373
Married/Living with Partner	2308 (67.58)	2310 (67.52)		
Widowed/Divorced/Separated	1047 (24.57)	1047 (24.54)		
Never married	327 (7.84)	328 (7.94)		
PIR, n (%)			χ^2=2.10	0.351
≤1.3	1091 (21.66)	1221 (21.99)		
1.3~3.5	1316 (37.67)	1467 (37.44)		
>3.5	923 (40.67)	997 (40.57)		
Drinking, n (%)			χ^2=4.85	0.089
No	1205 (29.84)	1268 (29.97)		
Less than once a week	1594 (48.47)	1700 (48.74)		
Greater than or equal to once a week	698 (21.69)	717 (21.29)		

CKD, n (%)			χ^2=0.00	0.967
No	2622 (75.15)	2654 (75.15)		
Yes	1020 (24.85)	1031 (24.85)		
Family history of CVD, n (%)			χ^2=0.57	0.451
No	3085 (83.52)	3168 (83.61)		
Yes	508 (16.48)	517 (16.39)		

PIR=poverty to income ratio; CKD=chronic kidney disease; CVD=cardiovascular disease.
